# Gene Expression Patterns of Osteopontin Isoforms and Integrins in Malignant Melanoma

**DOI:** 10.3389/pore.2022.1610608

**Published:** 2022-08-24

**Authors:** Krisztina Jámbor, Viktória Koroknai, Tímea Kiss, István Szász, Péter Pikó, Margit Balázs

**Affiliations:** ^1^ Doctoral School of Health Sciences, University of Debrecen, Debrecen, Hungary; ^2^ Department of Public Health and Epidemiology, Faculty of Medicine, University of Debrecen, Debrecen, Hungary; ^3^ ELKH-DE Public Health Research Group, University of Debrecen, Debrecen, Hungary

**Keywords:** gene expression, melanoma progression, osteopontin, osteopontin splice variants, integrins

## Abstract

Osteopontin (OPN) is a multifunctional glycoprotein that physiologically interacts with different types of integrins. It is considered to be a possible prognostic biomarker in certain tumor types; however, various splicing isoforms exist, which have not been investigated in melanoma. We aimed to define the relative expression pattern of five *OPN* isoforms and clarify the prognostic significance of the splice variants in melanoma. We also aimed to investigate the expression pattern of eight integrins in the same tumors. Gene expression analyses revealed that the relative expression of *OPNa, OPNb*, and *OPNc* is significantly higher in metastatic tumors compared to primary lesions (*p* < 0.01), whereas the expression of *OPN4* and *OPN5* was low in both. The more aggressive nodular melanomas had higher expression levels compared to the superficial spreading subtype (*p* ≤ 0.05). The relative expression of the eight tested integrins was low, with only the expression of *ITGB3* being detectable in nodular melanoma (Median_log2_ = 1.274). A positive correlation was found between Breslow thickness and the expression of *OPNc* variant, whereby thicker tumors (>4 mm) had significantly higher expression (*p* ≤ 0.05). The Breslow thickness was negatively correlated with the expression of *OPN4*, and similarly with *ITGA2*. *OPNc* also exhibited significant positive correlation with the presence of metastasis. Our data show that high expression of *OPNa*, *OPNb*, and especially *OPNc* and low expression of *OPN4* and *ITGA2* are associated with an advanced stage of tumor progression and poor prognosis in melanoma.

## Introduction

Osteopontin (OPN or SPP1) is a multifunctional glycoprotein that physiologically interacts with different types of integrins. OPN is considered to be a possible prognostic biomarker in certain tumor types including malignant melanoma (1, 2). Depending on the intracellular or extracellular localization, the expression of OPN is closely related to tumor proliferation, invasion, metastasis, and tumor microenvironment formation (3). These multifunctional biological roles are probably associated with the capacity for OPN to interact with different molecules, including cell surface receptors such as integrin and cluster of differentiation (CD44), intracellular signaling molecules, calcium, and heparin (4). OPN possesses three critical integrin binding sequences: the well conserved RGD domain (arginine–glycine–aspartic acid) that facilitated the interaction of OPN with αv integrins (especially αvβ1, αvβ3, and αvβ5); the SVVYGLR (serine–valine–valine–tyrosine–glutamate–leucine–arginine) domain, which may bind to α4β1, α4β7, and α9β1 integrins; and the ELVTDFP sequence on the N-terminal, which can bind α4β1 (5). The OPN protein is a member of the SIBLING (small integrin-binding ligand N-linked glycoprotein) family, whose members interact with CD44 and integrins through the characteristic domain. OPN is a secreted protein; however, the existence of a nonsecreted intracellular form (iOPN) has also been reported, which can be localized in the cytoplasm and nucleus and has a slightly different function than secreted OPN (sOPN) (6–8). OPN takes part in several normal physiological processes (vascularization, immune responses, inflammation, tissue remodeling, and cell adhesion) but also influences numerous aspects of tumorigenesis and metastasis (cell survival, proliferation, adhesion, migration, and invasion) (9, 10). Through the connection with integrins and CD44, OPN influences the PI3K/AKT signaling pathway, resulting in NF-κB-mediated cell proliferation and survival. Moreover, the interaction of OPN with αvβ3 integrin, in particular, affects the Ras/Raf/MEK/ERK signaling pathway and enhances the metastatic phenotype of several cancer cell types (11).

The primary transcript of the *OPN* gene is subject to alternative splicing, generating five splice isoforms: *OPNa, OPNb, OPNc, OPN4* and *OPN5* (3). The splicing variants differ in their gene structure: *OPNa* can be considered as the full-length isoform containing seven exons, *OPNb* lacks exon 5, and *OPNc* lacks exon 4. Both exon 4 and exon 5 are missing from *OPN* transcript variant 4, and, interestingly *OPN5*, has all seven exons supplemented with an extra exon derived from the retention of a portion from the intron 3 in the canonical isoform (12). Their translation results proteins called OPNa (314 amino acids), OPNb (300 amino acids), and OPNc (284 amino acids), which are widely studied and functionally well characterized, whereas OPN4 (273 amino acids) and OPN5 (327 amino acids) have only been recently identified (12–14). The splice variants are abnormally expressed in different types of tumors (3, 12). The high expression level of OPNc is indicative of adverse outcomes (nodal involvement, metastasis, and recurrence) in breast cancer, whereas the overexpression of the OPNa splice variant was observed in connection with the tumor growth of lung cancer cells, and OPNa was found to have a key role in thyroid cancer tumor progression (15, 16).

Recently, OPN has received increasing attention, with several studies investigating its role as a potential biomarker in different cancers, including melanoma (17–19). We previously observed, in our high-throughput microarray-based gene expression study, that ulcerated melanomas exhibit 6-fold higher expression of the *SPP1/OPN* gene compared to non-ulcerated melanomas (20). These results were also validated by qRT-PCR showing that elevated *OPN* mRNA expression is significantly associated with unfavorable prognostic parameters such as late stages (Clark stages IV–V), elevated Breslow thickness (≥4.00 mm), and ulcerated tumor surface (21). However, the expression patterns and the role of the *OPN* variants have not yet been described in human malignant melanoma. The expression of *OPN* splice variants in nonmelanoma skin cancers has only recently been investigated (22).

The aim of our study was to characterize the relative gene expression levels of *OPN* isoforms and clarify the prognostic significance of the five splice variants in primary and metastatic malignant melanoma. We also aimed to investigate the expression patterns of different integrins (*ITGA2*, *ITGA3*, *ITGA5*, *ITGA6*, *ITGA9*, *ITGAV*, *ITGB1*, and *ITGB3*) and assess their potential correlation with the clinicopathological parameters and osteopontin in the same tumors.

## Materials and Methods

### Melanoma Tissue Samples

Melanoma tissues were obtained from the Department of Dermatology at the University of Debrecen, Hungary. This study was approved by the Regional and Institutional Ethics Committee of the University of Debrecen Clinical Center [ETT TUKEB 26364-1/2012/EKU (449/PI/12)] and was carried out according to all relevant regulations. Written informed consent was obtained from the patients. Lesions were diagnosed on the basis of formalin-fixed paraffin-embedded tissue sections stained with hematoxylin–eosin. A total of 31 primary and 10 metastatic melanoma samples were analyzed. The metastatic samples were derived from different patients other than the primary tumors. The follow up period of patients was 5 years. The clinical–pathological parameters of the melanoma tissue samples are summarized in Table 1.

**TABLE 1:** Clinicopathological parameters of melanoma tissue samples.

Variables	n
Primary melanoma samples (*n* = 31)	
SSM^a^	21
NM^b^	10
Gender	
Male	16
Female	15
Age (years)	
20–50	7
>50	24
Breslow thickness (mm)^c^	
<2.00	8
2.01-4.00	14
>4.00	9
Clark’s level	
II–III	13
IV–V	17
n.d.	1
Ulceration	
Absent	18
Present	13
Localisation	
Trunk	15
Extremities	13
Head	3
Metastasis formation^d^	
Non-metastatic	13
Metastatic	18
Melanoma metastases (*n* = 10)	
Male	7
Female	3
Age (years)	
20–50	2
>50	8
Localization	
Regional lymph node	4
Regional (sub)cutaneous	3
Distant	3

a SSM, superficial spreading melanoma.

b NM, nodular melanoma.

c Thickness categories based on the current melanoma staging system.

d Patients with follow-up periods of 5 years were included into the study.

Before RNA isolation all melanoma tissue samples were examined for the content of tumor cells and the adjacent normal tissues were removed to ensure that normal cell contamination will not influence the results. The tumor cell content of tissues analysed were ≥80% for each sample. The total RNA was isolated from frozen melanoma tissues using the RNeasy Plus Mini Kit (Qiagen GmbH, Hilden, Germany) according to the manufacturer’s protocol. The concentration and the quality of the RNA was determined using NanoDrop ND-1000 UV–vis Spectrophotometer V3.3.0 (NanoDrop Technologies, Wilmington, DE, United States). The absorbance ratios of 260 nm/280 nm of all RNA samples were 1.8 or above and the 260 nm/230 nm ratios were 2.0 or above. cDNA synthesis of 600 ng total RNA was performed using a High Capacity cDNA Reverse Transcription Kit (Applied Biosystems, Foster City, CA, United States) according to the manufacturer’s protocol.

### qRT-PCR

Gene expression levels of OPN splice variants (*OPNa*, *OPNb*, *OPNc*, *OPN4*, and *OPN5*) and integrins (*ITGA2*, *ITGA3*, *ITGA5*, *ITGA6*, *ITGA9*, *ITGAV*, *ITGB1*, and *ITGB3*) were determined by real-time PCR using Xceed qPCR Probe 2x Mix Hi-ROX (Institute of Applied Biotechnologies, Prague, Czech Republic) and a LightCycler® 480 Instrument II (Roche Diagnostics Nederland BV, Almere, Netherlands). Each reaction contained 15 ng cDNA and was run on the LightCycler® 480 instrument. Conditions for real-time PCR included the following steps: preactivation: 95°C 1 min; followed by 45 cycles of the following program: 95°C 5 s (denaturation), annealing 55–62°C for 10 s (specific annealing temperature and the sequence of each primer can be found in Table 2), extension 72°C 15 s, cooling at 40°C for 30 s; finished by melting curve analysis. Primers were obtained from Life Technologies. To analyze qRT-PCR data, cyclophilin A (CYPA) was used as a reference gene and the Livak method (2^−ΔΔCT^ equation) was applied (23). Pooled nevi (*n* = 8) was used as a normalization control for the melanoma tissue samples. Statistical analysis was performed using IBM SPSS (Statistical Package for Social Sciences) Statistics for Windows, version 25.0 (IBM Corp., Armonk, NY, United States).

**TABLE 2:** Primer sequences of OPN splice variants, reference gene and integrins used for qRT-PCR.

Gene	Nucleotide sequence	T annealing (°C)
OPNa F	ATC​TCC​TAG​CCC​CAC​AGA​AT	55
OPNa R	CAT​CAG​ACT​GGT​GAG​AAT​CAT​C	
OPNb F	ATC​TCC​TAG​CCC​CAG​AGA​C	55
OPNb R	AAA​ATC​AGT​GAC​CAG​TTC​ATC​AG	
OPNc F	TGA​GGA​AAA​GCA​GAA​TGC​TG	57
OPNc R	GTC​AAT​GGA​GTC​CTG​GCT​GT	
OPN4 F	GGAAAAGCAGACCCTTCC	55
OPN4 R	CAT​ATG​TGT​CTA​CTG​TGG​GG	
OPN5 F	AAC​AAA​TGG​GCA​TTG​TCC​CC	59
OPN5 R	GCA​GTC​TAA​TTG​CAG​TGA​CCC	
CYPA F	CTC​GAA​TAA​GTT​TGA​CTT​G	60
CYPA R	CTA​GGC​ATG​GGA​GGG​AAC​A	
ITGA2 F	CAC​AAA​GAC​ACA​GGT​GGG​GT	62
ITGA2 R	TGG​GAT​GTC​TGG​GAT​GTT​GC	
ITGA3 F	GCC​CAC​AAG​GAT​GAC​TGT​G	60
ITGA3 R	GCT​GGT​CTT​CTG​ACC​CTG​A	
ITGA5 F	GAG​CAA​GAG​CCG​GAT​AGA​GG	55
ITGA5 R	CTG​CTC​CCC​AAA​CAC​TTC​CA	
ITGA6 F	AAA​CTG​CGT​CCC​ATT​CCC​A	60
ITGA6 R	TGT​CGT​CTC​CAC​ATC​CCT​C	
ITGA9 F	CGG​TAC​ACC​TAC​CTG​GGC​TA	58
ITGA9 R	AAA​CCT​TGC​CGA​TGC​CTT​TG	
ITGAV F	AAT​GTT​GTG​CCG​GAT​GTT​TCT​T	58
ITGAV R	CGG​GTA​GAA​GAC​CAG​TCA​CAT	
ITGB1 F	CCA​AAT​GGG​ACA​CGG​GTG​AA	58
ITGB1 R	GTG​TTG​TGG​GAT​TTG​CAC​GG	
ITGB3 F	CCT​CAT​CAC​CAT​CCA​CGA​CC	62
ITGB3 R	GTT​GTT​GGC​TGT​GTC​CCA​TT	

### Statistical Analysis

The expression data were analyzed in the melanoma tissue samples classified by histological tissue subtype, Breslow thickness of the primary tumors, and Clark staging. The following Breslow thickness groups were applied based on the current melanoma staging system: tissues from tumors with less than 2 mm thickness (*n* = 8), between 2 and 4 mm (*n* = 14), and tumors with more than 4 mm thickness (*n* = 9) (24). The Kruskal–Wallis H test was used to determine the significant differences of expression data between more than two groups. A two-sided Mann–Whitney *U* test was applied to reveal significant differences between the expression data of two certain tissue sample groups. Stepwise regression analysis was applied to select those OPN variants and integrins whose expression demonstrate a relationship with the Breslow thickness (as a continuous variant) independently of each other and without collinearity. Linear regression (adjusted for age and sex) was carried out to determine which of these variants were significantly associated with Breslow thickness. Primary melanomas were also grouped according to whether the patient was diagnosed with metastasis or did not develop metastasis during the follow-up (5 years) period. The expression data were analyzed by logistic regression. *p* < 0.05 was considered to indicate statistically significant differences in each case.

## Results

### Relative Gene Expression of OPN Splicing Isoforms and Their Comparison in Melanoma Tissue Subtypes

**FIGURE 1:**
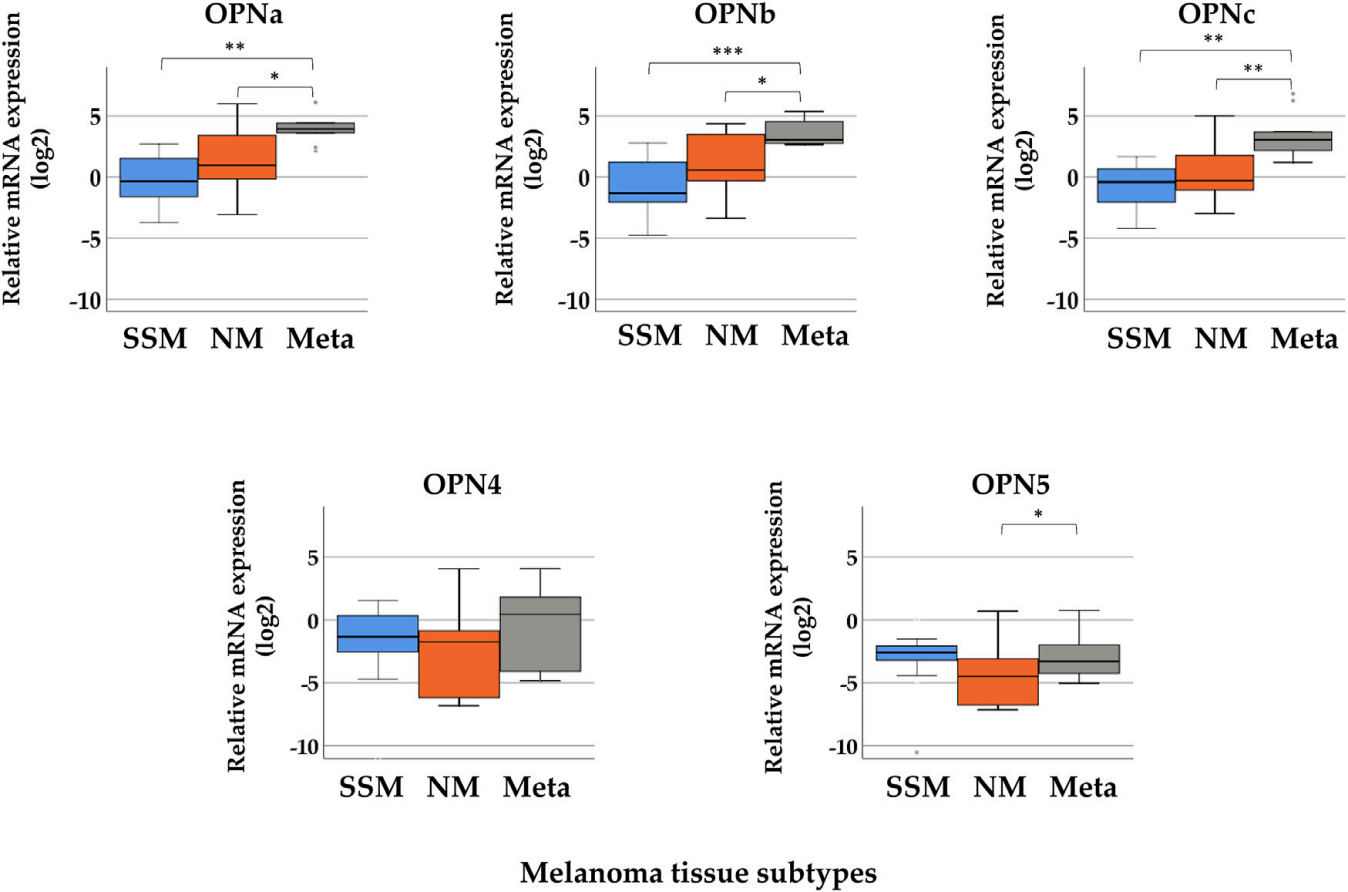
Relative mRNA expression levels of osteopontin isoforms (*OPNa*, *OPNb*, *OPNc*, *OPN4*, and *OPN5*) in distinct pathological subgroups of malignant melanoma: superficial spreading melanoma (SSM, *n* = 21), nodular melanoma (NM, *n* = 10), and melanoma metastasis (Meta, *n* = 10) tissue samples. Asterisks indicate significant differences: *p < 0.05; **p < 0.01; ***p < 0.001.

The median gene expression values determined for *OPN* splicing isoforms in SSM, NM, and melanoma metastasis tissue samples are summarized in Supplementary Table S1. In Figure 1, boxplots show the relative expression of the five splice variants (*OPNa*, *OPNb*, *OPNc*, *OPN4*, and *OPN5*) in superficial spreading melanomas, nodular melanomas, and melanoma metastasis. The highest expression of *OPNa* (median = 3.925), *OPNb* (median = 3.043), and *OPNc* (median = 3.060) was detected in melanoma metastasis, which significantly differed when compared to the nodular and superficial tissue samples (*p* ≤ 0.05). The relative expression levels of *OPN4* and *OPN5* were very low in all pathological subtypes, and they were downregulated relative to nevi (except in the case of *OPN4* in metastasis). A significant difference in the expression of *OPN5* was observed only between nodular and melanoma metastasis tissues (*p* ≤ 0.05).

### Comparison of the Relative Gene Expression of OPN Splice Variants in Tissues Classified by Breslow Thickness and Clark Stages in Primary Tumor Samples

**FIGURE 2:**
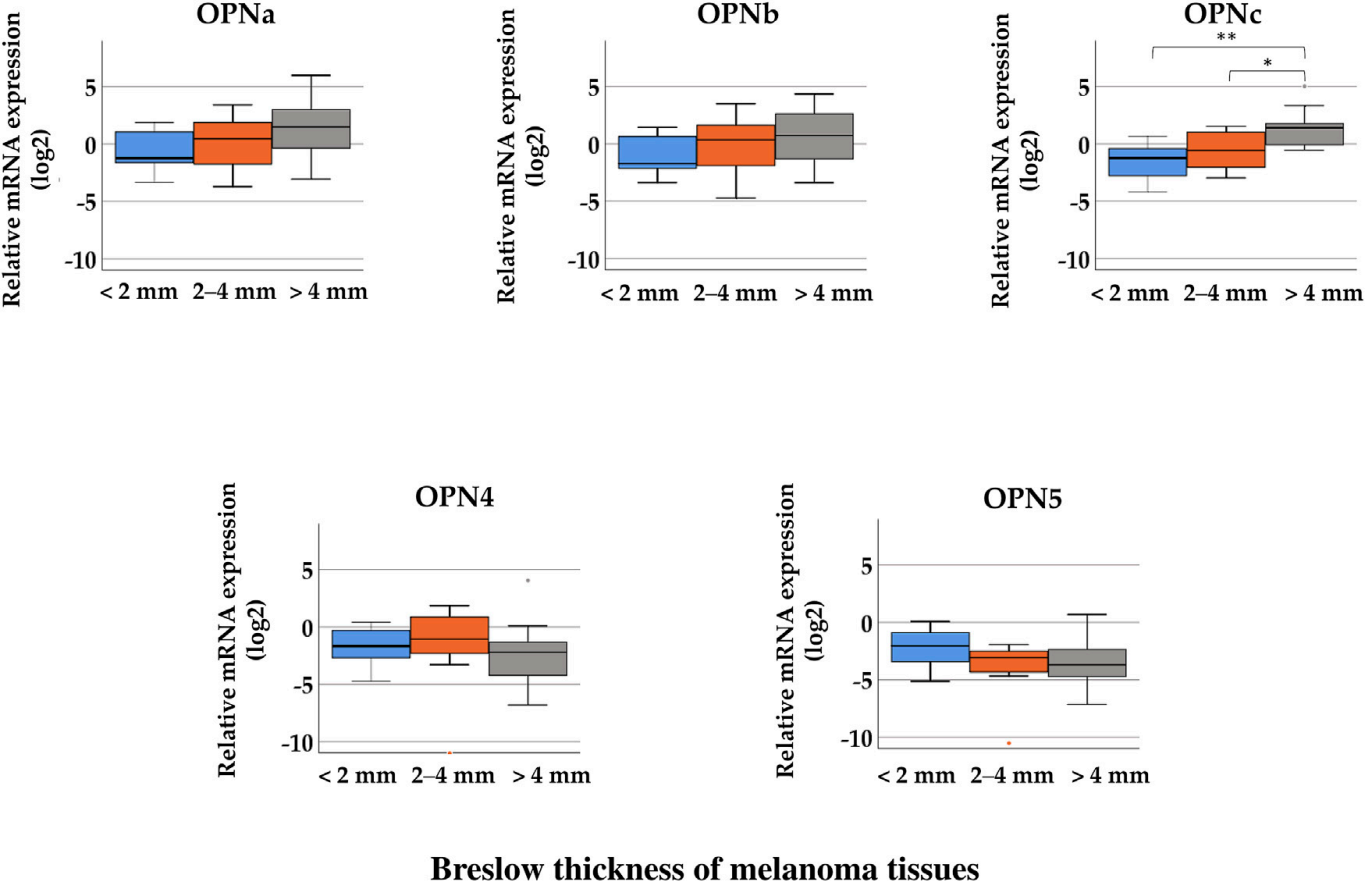
Comparison of the relative mRNA expression of osteopontin isoforms (*OPNa*, *OPNb*, *OPNc*, *OPN4*, and *OPN5*) in malignant melanoma tissue samples from tumors with different Breslow thicknesses: < 2 mm (*n* = 8), 2–4 mm (*n* = 14), and >4 mm (*n* = 9). Significant differences (*p ≤ 0.05; **p ≤ 0.01; ***p ≤ 0.001 Mann–Whitney test, Kruskal–Wallis test) are indicated by asterisks.

Investigating the relative gene expression of the OPN isoforms in the three thickness groups, the Kruskal–Wallis H test showed significant differences in the relative expression of *OPNc* between the groups (<2 mm (median = −1.24), 2–4 mm (median = −0.57), and >4 mm (median = 1.40); *p* = 0.008). When comparing two groups with each other, tissues samples with >4 mm Breslow thickness showed significantly higher relative expression of *OPNc* than samples with 2–4 mm thickness (Mann–Whitney *U* test *p* = 0.023), and tissues with less than 2 mm exhibited the lowest expression (Figure 2). This trend of elevation can also be observed in the expression of *OPNa* and *OPNb* variants as the thickness increases; however, these differences were not considered significant. The mRNA expression of *OPN4* and *OPN5* did not show significant association with Breslow thickness. Supplementary Table S2 summarizes the median values of each group. We did not find significant association between the relative gene expression levels of the different OPN splice variants and Clark stages.

### Relative Gene Expression of Integrins and Their Comparison in Pathological Subgroups of Melanoma Tissues

**FIGURE 3:**
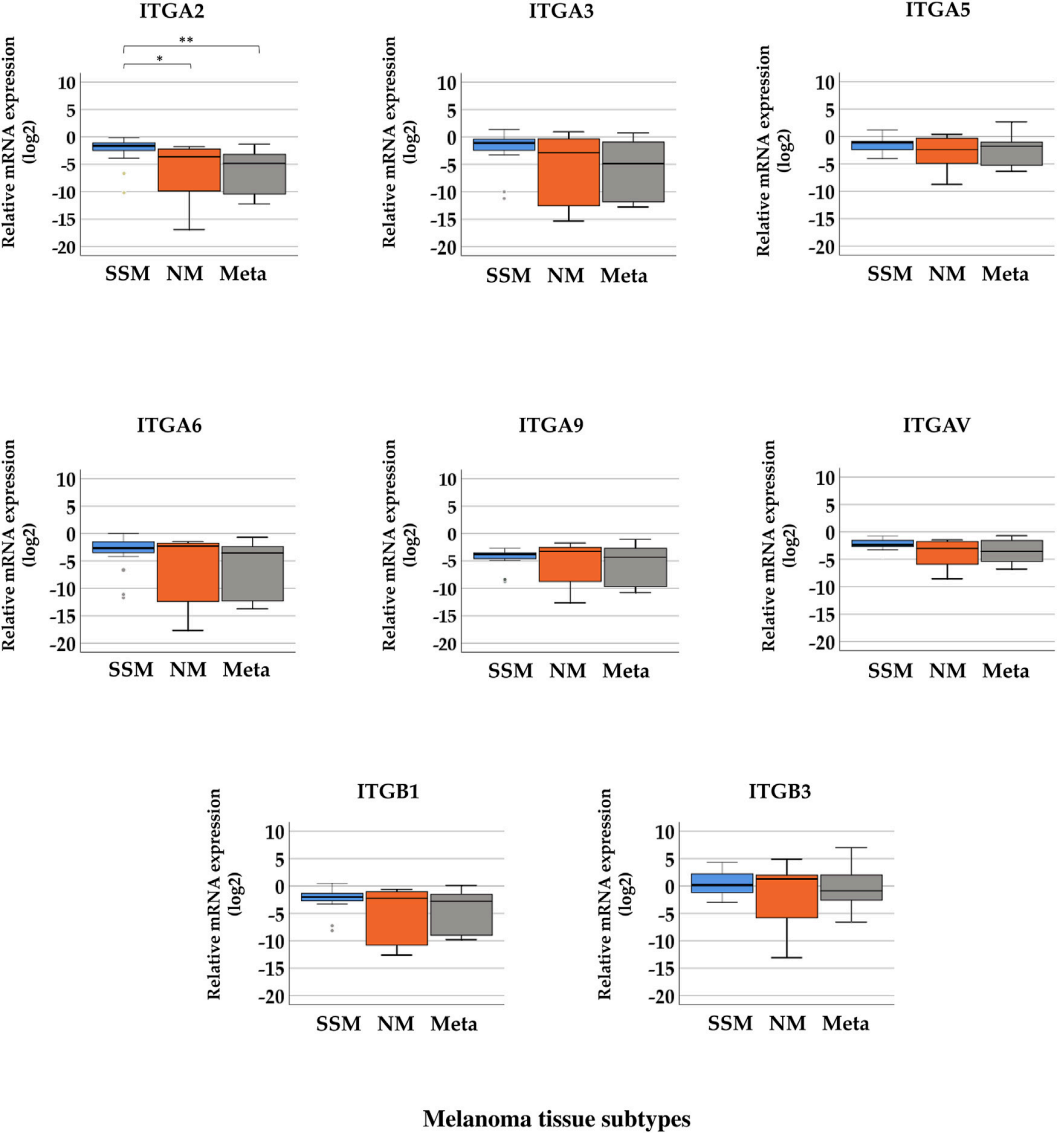
Relative mRNA expression levels of integrin (*ITGA2*, *ITGA3*, *ITGA5*, *ITGA6*, *ITGA9*, *ITGAV*, *ITGB1*, and *ITGB3*) genes in distinct pathological subgroups of malignant melanoma: superficial spreading melanoma (SSM, *n* = 20), nodular melanoma (NM, *n* = 9), and melanoma metastasis (Meta, *n* = 10) tissue samples. Asterisks indicate significant differences (*p < 0.05; **p < 0.01; ***p < 0.001).

Boxplots of the expression levels of the eight integrin genes in subgroups of melanoma tissue samples are presented in Figure 3. Excluding *ITGA2*, seven integrins exhibited no significant differences in gene expression between different tissue subtypes. A significant difference in the gene expression level of *ITGA2* was found in SSM tissue samples (median = −1.622) compared with NM (median = −3.630) and melanoma metastasis (median = −4.807) tissue samples. (Kruskal–Wallis H test, *p* = 0.001; Mann–Whitney *U* test, *p* = 0.002). However, the expression of the eight tested integrin genes was, overall, extremely low (with negative values for medians indicating downregulation) except *ITGB3*, which showed measurable values without significant differences in expression (median values: M_SSM_ = 0.168, M_NM_ = 1.274, and M_Metastasis_ = −0.863). Supplementary Table S3 summarizes the median values of relative gene expression data in each melanoma tissue subgroup.

### Relationship Between the Relative Gene Expression of Integrins and the Clinicopathological Data (Breslow Thickness and Clark Staging) of Primary Melanoma Tissues

**FIGURE 4:**
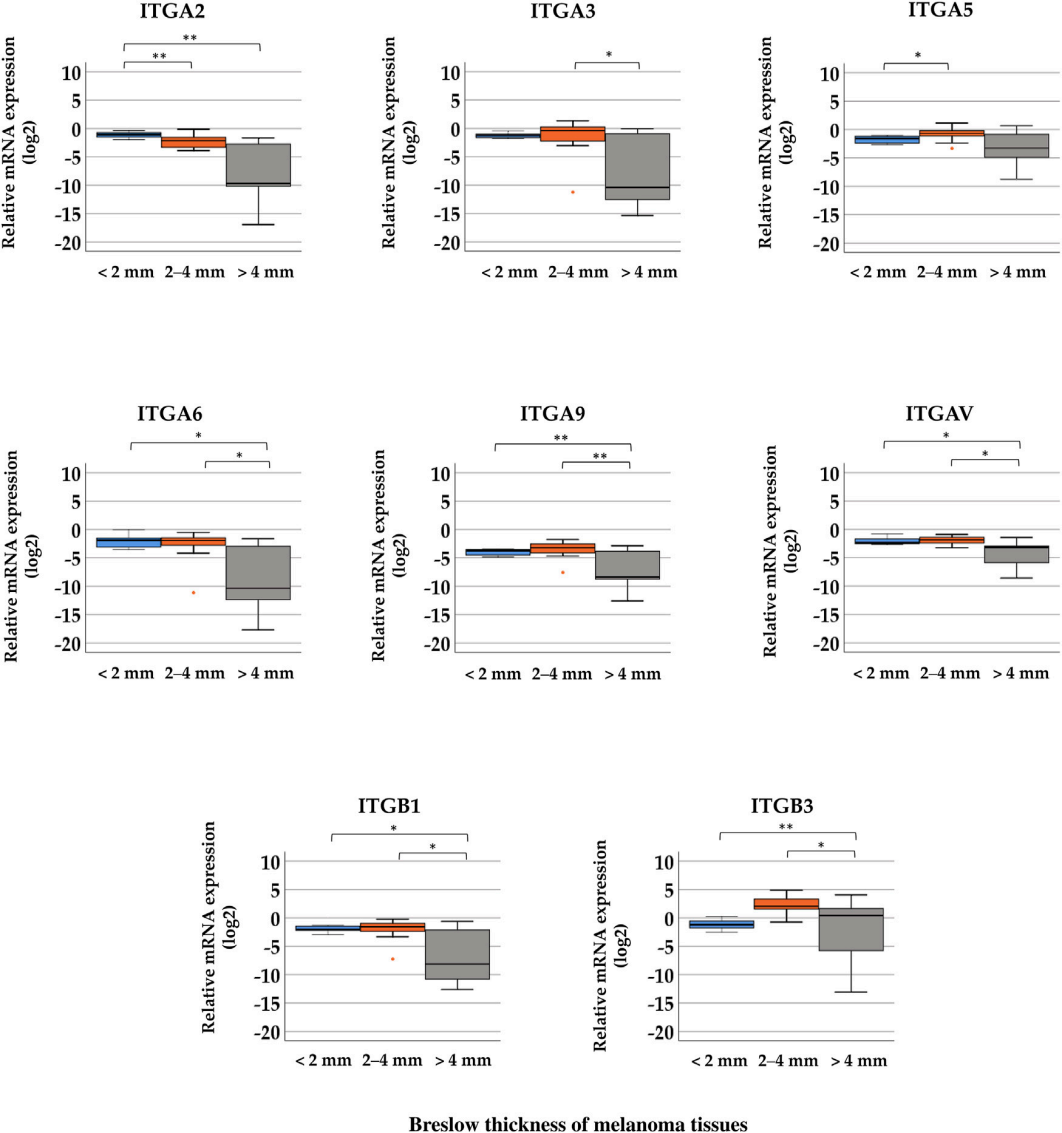
Comparison of relative mRNA expression levels of integrin (*ITGA2*, *ITGA3*, *ITGA5*, *ITGA6*, *ITGA9*, *ITGAV*, *ITGB1*, and *ITGB3*) genes in melanoma tissue samples grouped by distinct Breslow thickness: <2 mm (*n* = 8), 2–4 mm (*n* = 12), and >4 mm (*n* = 9). Significant differences (*p ≤ 0.05; **p ≤ 0.01; ***p ≤ 0.001 Mann–Whitney test, Kruskal–Wallis test) are indicated by asterisks.

In the cases of *ITGA2*, *ITGA6*, *ITGA9*, *ITGAV*, *ITGB1*, and *ITGB3*, significant differences were observed between the three Breslow thickness groups (Figure 4, Kruskal–Wallis test, *p* < 0.05). Tumor samples with more than 4 mm thickness exhibited significantly lower relative expression levels of *ITGA3, ITGA6, ITGA9, ITGAV*, and *ITGB1* than samples belonging to the 2–4 mm Breslow thickness category (Mann–Whitney test, *p* < 0.05). *ITGB3* exhibited significantly higher relative expression in tissues with 2–4 mm and >4 mm Breslow thickness, and was the highest in the group with 2–4 mm thickness. Median values can be found in Supplementary Table S4.

Tissue samples of primary tumors were also differentiated according to Clark stages: earlier stages (II–III, *n* = 13), and later stages (IV–V, *n* = 17) Supplementary Figure S1 shows that a significant difference was observed only in the case of *ITGA2*: tissues with a later Clark stage (IV–V) exhibited lower mRNA expression (mean = −1.35) than samples with earlier stage (II–III) (mean = −3.57) (Mann–Whitney *U* test, *p* < 0.005).

### Correlation of the Relative Expression of Osteopontin Variants and Integrins

The gene expression data of OPN variants and integrins analyzed with Spearman’s correlation revealed positive correlations of expression between *OPN4* and most of the integrins: *ITGB3* (*r* = 0.604), *ITGA5* (*r* = 0.530), *ITGA9* (*r* = 0.530), *ITGAV* (*r* = 0.520), *ITGB1* (*r* = 0.590), *ITGA3* (*r* = 0.585), and *ITGA6* (*r* = 0.500) (*p* < 0.01). Positive correlations of expression were also observed between *OPN5* and *ITGA2* (*r* = 0.447), *ITGAV* (*r* = 0.504) (*p* < 0.01), *ITGB1* (*r* = 0.348), *ITGA3* (*r* = 0.361), and *ITGA6* (*r* = 0.406) (*p* < 0.05). Negative correlations of expression were observed between *ITGA2* and *OPNa* (*r* = −0.480), *OPNb* (*r* = −0.416), and *OPNc* (r = −0.540) (*p* < 0.01) and between *ITGA6* and *OPNc* (*r* = −0.392) (*p* < 0.05). A table with the results of the Spearman’s rho correlation can be found in Supplementary Table S5 and graphs of significantly correlating variables in Supplementary Figures S2–S5.

### Correlation of OPN Variants and ITGs With Breslow Thickness (As a Continuous Outcome in mm) Based on the Results of Stepwise and Linear Regression Analysis

As a result of stepwise regression analysis, the expression of two osteopontin variants, *OPNc* and *OPN4*, and two integrins, *ITGA5* and *ITGA2*, was determined to be linked with Breslow thickness independently. The linear regression analysis of these variants revealed that their relationship with Breslow thickness was significant; however, while the beta value of *OPNc* was 0.217, indicating a positive correlation, the negative beta value of *OPN4*, −0.762, indicates a negative correlation with Breslow thickness. In the case of integrins, *ITGA2* had a significant negative correlation (*β* value = −23.061), but *ITGA5* showed a significant positive correlation (*β* value = 2.697) with Breslow thickness. A 0.1 unit (2^−ΔΔCt^) decrease in *ITGA2* expression is associated with 2.3 mm increase of Breslow thickness. See Table 3 for more details.

**TABLE 3:** Linear regression analyses of the gene expression of OPN splice variants and integrins with the Breslow thickness of primary melanomas.

Osteopontin isoforms	β	95% CI	p-value
OPNa	0.115	−0.011–0.242	0.071
OPNb	0.172	−0.069–0.414	0.155
OPNc	**0.217**	**0.112–0.322**	**<0.001**
OPN4	−**0.762**	−**1.343–(**−**0.181)**	**0.012**
OPN5	−3.030	−8.237–2,176	0.242
**Integrins**	**β**	**95% CI**	**p-value**
ITGA2	−**23.061**	−**30.158–(**−**15.964)**	**<0.001**
ITGA3	−2.338	−6.861–2.185	0.296
ITGA5	**2.697**	**0.956–4.438**	**0.004**
ITGA6	−9.079	−20.317–2.158	0.108
ITGA9	−14.345	−41.876–13.185	0.292
ITGAV	−15.465	−30.958–0.046	0.051
ITGB1	−5.086	−13.612–3.440	0.229
ITGB3	0.023	−0.099–0.146	0.7

β is a regression coefficients which indicates the direction and size of effect between variables. CI: confidence interval. Bold values indicates significant associations.

### Logistic Regression: Relative Expression of OPN Variants and Presence of Metastasis

The results of logistic regression show that *OPNc* expression (log2 transformed data) is significantly positively correlated with the presence of metastasis (OR = 1.931, *p* = 0.044) (Table 4), whereas *OPN4* was not significantly correlated (OR = 0.962, *p* = 0.773) with the presence of metastasis ***.*** Integrins did not show significant correlation with the presence of metastasis.

**TABLE 4:** Logistic regression analyses of the gene expressions OPN splice variants and integrins in relation of metastasis formation of malignant melanoma.

Osteopontin isoforms	OR	95% CI	p-value
OPNa	1.223	0.855–1.749	0.270
OPNb	1.070	0.759–1.507	0.699
OPNc	**1.931**	**1.018–3.661**	**0.044**
OPN4	0.962	0.743–1.248	0.773
OPN5	1.144	0.867–1.509	0.341
**Integrins**	**OR**	**95% CI**	**p-value**
ITGA2	0.685	0.348–1.349	0.274
ITGA3	0.411	0.135–1.250	0.117
ITGA5	0.889	0.396–1.996	0.776
ITGA6	0.560	0.234–1.337	0.191
ITGA9	0.675	0.407–1.120	0.129
ITGAV	0.280	0.065–1.207	0.088
ITGB1	0.333	0.085–1.305	0.115
ITGB3	0.836	0.642–1.088	0.182

OR, odds ratio; CI, confidence interval. Bold values indicates significant associations.

## Discussion

Osteopontin (OPN), a multifunctional protein, has been widely studied as a promising biomarker in various types of tumors for monitoring tumor progression, invasion, metastasis formation and drug resistance (13, 25). The association between the aberrant expression of osteopontin and melanoma invasion, metastasis formation, and radio/drug resistance has been recently described (1, 3, 25–27). The biological functions of tumor-associated gene products are extensively regulated via pre- and posttranscriptional modifications, resulting in alternative splicing of OPN. Alternative splicing of various mRNA products of a single gene is a critical mechanism for generating proteomic diversity. OPNa, OPNb, and OPNc variants were first described in glioma, and the two additional splice variants (OPN4 and OPN5) were found in esophageal adenocarcinomas and glioblastomas (28). Because the different splice variants of OPN are associated with different types of cancers, it is assumed that the isotypes may have different functions. Osteopontin isoforms display functional heterogeneity and cell and tissue specificity, which still poses challenges while providing opportunities for novel diagnostic, prognostic, and therapeutic strategies (13).

Most of the studies investigating the role of OPN splice variants have focused on the expression of *OPNa*, *OPNb*, and *OPNc*; however, the data in distinct tumor types are conflicting, and the functional heterogeneity of the variants serves as motivation for researchers to define the role of OPN splice variants in each type of cancer. In addition, at present, no data on the isoform expression patterns in malignant melanoma are available. Therefore, in this study, our primary aim was to study the expression profile of five *OPN* splice variants (*OPNa*, *OPNb*, *OPNc*, *OPN4*, and *OPN5*) in different types of malignant melanoma tissues and investigate the association with the clinicopathological features of tumor tissues. In addition, because OPN signaling occurs through different integrin receptors, we also aimed to examine the relative mRNA expression profile of eight integrins (*ITGA2*, *ITGA3*, *ITGA5*, *ITGA6*, *ITGA9*, *ITGAV*, *ITGB1*, and *ITGB3*) and analyze the correlation of the relative expression of osteopontin variants and integrins in the same melanoma samples.

We found elevated relative mRNA expression of *OPN* variants (*OPNa*, *OPNb*, and *OPNc*) in nodular melanomas and melanoma metastasis compared to samples of superficial spreading melanoma. The significant increase of these isoforms in the more advanced stages indicates that they may contribute to tumor progression and worse outcome, since lower survival rates were observed in NM and metastatic melanoma patients (29). The significant elevation of *OPNc* expression in thicker melanoma tissue samples suggests that it is associated with increasing Breslow thickness. The elevating tendencies of the expression levels of the other variants were similar; however, none were found to be significant in the comparison. Our further statistical analysis also confirmed the significant positive association of *OPNc* with Breslow thickness: a one-unit increase in *OPNc* expression is associated with a 0.217 increase in Breslow thickness. Based on our observations, it is possible that *OPNc* expression has a crucial role in melanoma tumor progression, and that elevated levels of this variant can contribute to progression toward advanced stages of disease and even the induction of metastasis formation, and it can thus serve as an indicator of an aggressive phenotype.

The expression of *OPN* splice variants has already been investigated in hematological malignancies, thyroid tumors, and gastric cancers (18, 30). Wide variations were observed in the expression patterns and the predominantly expressed variant(s) depending on the tumor type; therefore, it is difficult to establish a possible universal nature or profile. It could be convenient to establish the expression profile of each splice variant in each distinct cancer type. To date, it has been observed that the OPNa mRNA levels were significantly associated with high TNM staging and unfavorable clinical outcomes in gastric cancer; moreover, *OPNa* and *OPNb* are correlated with short overall and disease-free survival of patients (30). It was pointed out that *OPNc* expression is also associated with advanced stage, tumor recurrence, and metastasis formation; thus, *OPNc* is considered to be a promising prognostic factor in breast cancer (15). In our results, the elevated expression of the *OPNc* variant in an advanced stage of primary melanoma and melanoma metastasis samples in addition to its significant correlation with the presence of metastasis indicates the importance of *OPNc* in melanoma progression, metastasis formation, and the relationship with the aggressive phenotype, which justifies its further investigation at multiple levels as a promising prognostic biomarker in malignant melanoma. Since no specific antibodies against these splice variants are currently available (except for *OPNc* from Gallus Immunotech Inc. which is a polyclonal antibody), the future development of the OPN isotype antibodies could also be an important step forward in the characterization of these variants. Moreover, investigating the specific role of each splice variant in melanoma progression could bring us closer to developing potential targetable molecules in melanoma therapy.

While the exact role of *OPNc* overexpression on tumor progression is unknown, there are several possible explanations for its association with metastasis. Since *OPNc* variant lacks exon 4, which contains the target sequence for transglutaminase, it lacks an important domain for calcium induced aggregation and transglutamination (31). *OPNc*, unlike the other isoforms, cannot form polymeric complexes. This might be the essential reason for the pathological role of *OPNc* and it may not be cross-linked with extracellular matrix and thus it results cell migration. The full length OPN aggregates and enhances cell adhesion and therefore reduces dissemination of tumor cells, whereas *OPNc* promotes tumor invasion and metastasis formation because of its lack of aggregation (32). The non-aggregative nature of *OPNc* is in concert with the relative resistance to polymerization (33). On the other hand, *OPNc* can stimulate cell proliferation rates independently of growth factors, a feature of proteins typically involved in tumor progression (34). While numerous studies suggest that OPN plays a key role in mediating tumor progression and metastasis by regulating various pathways, very few data are available for the role of different OPN splice variants, the expression patterns of the OPN isoforms in malignant melanoma was first described in the present study. In order to discover the detailed functional role of *OPNc*, the 3D structure of the variant might be useful. It is an important step that the tertiary structure of *OPNc* was successfully predicted by Sivakumar and this predicted structure might be used for computational drug design of *OPNc* with respect to cancer prevention (31).

Aside from the three most frequently studied OPN variants, we also investigated the expression of *OPN4* and *OPN5* isoforms that were recently described (4, 35). Surprisingly, relative expression of *OPN4* and *OPN5* was low in primary as well as metastatic tissues. Though statistical analysis showed a significant difference in *OPN5* expression between nodular melanoma and melanoma metastasis histological subtypes, *OPN4* and *OPN5* were downregulated, and the median expression values did not exceed zero on the logarithmic scale (except in the case of *OPN4* in melanoma metastasis). Although significant differences were not found when comparing their expression between the sample groups for different Breslow thicknesses, logistic regression demonstrated a significant negative correlation between *OPN4* and Breslow thickness and conversely in the case of *OPNc*, which was positive correlated. Moreover, the analysis of the Spearman’s correlation between osteopontin splice variants and integrins in expression data revealed the positive correlation of *OPN4* expression with that of most of the integrins and the negative correlation of *ITGA2* expression with that of most of the osteopontins, suggesting that *OPN4* may have an expression profile more similar to that of the integrins than that of the osteopontins.

The expression of the *OPN4* and *OPN5* variants was previously investigated in esophageal adenocarcinoma tissue samples (4), where, unlike in our study, it was found that the expression of *OPN4* and *OPN5* was elevated in primary tumors when compared to normal and Barrett’s samples, and the isoforms were co-overexpressed. In another study, the expression of these two isoforms was found to be variable in most of the tested 7 cell lines (prostate tumor, ovarian cancer, B-cell precursor acute lymphoid leukemia, breast cancer, colorectal cancer, and thyroid and lung tumors) (31). Except for in the two breast cancer cell lines, *OPN4* and *OPN5* were found to be co-expressed in the other 5 cell lines, but the expression patterns differed from those of the previously characterized *OPNa, OPNb,* and *OPNc* variants (31). According to the study of Chou et al., besides the predominant expression of *OPNa* variant, *OPN4* was found to be minimally expressed in normal skin and nonmelanoma skin cancer, but *OPN5* exhibited higher expression in normal skin than *OPNb* and *OPNc*, and *OPN5* was more highly expressed in nonmelanoma skin cancer than *OPNc* (22). Taken together, the expression of *OPN4* and *OPN5* splice variants appears to vary widely in distinct tumor types, but in melanoma, they slightly have a different expression profile than the three predominant splice variants.

In connection with the expression profile of integrins, our results show that the relative mRNA expression levels of the investigated integrin genes are extremely low except in one case. Median values of the integrin expression levels in SSM, NM, and melanoma metastasis were equally below zero, which means they are downregulated compared to the nevus control. As the exception, *ITGB3* appeared to be upregulated in SSM and NM tissue samples. Moreover, when comparing integrin gene expression in melanoma tissues with distinct Breslow thickness, a significantly higher *ITGB3* expression was observed in thicker tissue samples (2–4 mm and > 4 mm) compared to tissues with lower Breslow thickness (<2 mm) (*p* = 0.004). This result is in accordance with the relevant literature: in malignant melanoma, upregulated expression of subunit β3 was found in the vertical growth phase, which was linked with disease progression and correlated with poor survival and lymph node and lung metastasis formation (36, 37).

Other previous *in vivo* and *in vitro* studies have also investigated altered integrin expression, summarized in detail by Arias-Mejias et al. (38). It was found that elevated expression of integrin β3 protein (the dimerized form is αvβ3 or αIIbβ3) in human melanoma cells and tissues was associated with tumor progression, organ-specific metastasis formation, disease recurrence, and decreased long-term survival (39, 40). In our earlier study we identified metastasis correlated genes, including many genes involved in signaling in the immune system (HLA antigens), cell adhesion and cell motility networks (40, 41). These networks involve genes such as that of integrins (*ITGA2*, *ITGA3*, *ITGA4*, *ITGA9*, *ITGB5* or *ITGB8*). Investigating the expression of these genes in metastatic primary melanomas and metastases, we found that *ITGA3* was downregulated in both regional and distant organ metastases compared to the metastatic primary lesions. In the present study, even the direction of the mRNA expression was similar for *ITGA3*, we observed significant decrease only for the *ITGA2* gene in the metastatic tumors. The inconsistency between the two investigations can be explained by the fact that the composition of primary tumor groups was different between the two studies.

In our case, interestingly, a significant difference between the histological subtypes was observed only in the expression of *ITGA2*; however, it displays a decreasing tendency in NM and metastasis samples compared to SSM. Relative expression of integrins in melanoma tissue samples with distinct Breslow thicknesses varies, six integrins (*ITGA3*, *ITGA6*, *ITGA9*, *ITGAV*, *ITGB1*, and *ITGB3*) out of the eight showed a significant difference between the 2–4 mm and the > 4 mm group (lowest expression in the thickest group), and again, expression of *ITGA2* showed a significant decrease as the thickness increased. Linear regression analysis indicated that while *ITGA5* had positive correlation with Breslow thickness (as a continuous variant) *ITGA2* was in strong significant inverse correlation with the Breslow thickness of the tumor. These results suggest that downregulation of *ITGA2* may be linked with tumor progression in malignant melanoma. This hypothesis seems to be supported by the results of *in vitro* studies with breast cancer mouse models, which suggest that integrin α2 might function as a metastasis suppressor (42). Moreover, decreased expression of the α2 subunit was found to be associated with more advanced status, such as higher tumor nodal status or presence of metastasis (41). Madamanchi et al. describe that certain other cancer types (prostate, colon, and lung cancer) also seem to be associated with reduced integrin α2β1, which is associated with tumor progression and metastasis. However, they also note that other cancer types were associated with high α2β1 integrin expression levels; hence, the exact biological role of this integrin is being heavily debated (43). Indeed, the relevance of the α2β1 integrin as a main regulator of metastasis in tumor cells was discovered only in recent years, and these findings appear to be controversial compared with the results of the previously mentioned studies. The crucial role of the α2β1 integrin has been determined in cancer types including melanoma, as it is responsible for regulating cell migration, survival, proliferation, and metastasis formation in the lung and liver (44, 45). Upregulation of α2β1 was found in highly metastatic melanoma compared to nonmetastatic or poorly metastatic cell lines, where it was associated with enhanced cell migration (38). Increased expression of α2β1 in malignant melanoma compared to benign tumors was found to stimulate angiogenesis and facilitate tumor growth (46).

Though acquiring a comprehensive understanding of integrin signaling is challenging, some possible reasons may explain the differences in the expression of the various integrins and their role in tumor progression. Single nucleotide polymorphisms may change the affinity of *ITGA2* for transcription factors, which can alter the transcription rate (47). Enhancing transcription–coactivator complex binding can increase *ITGA2* transcription. In addition, different posttranslational modifications, such as sialylation and glycosylation, can modify the role of integrins in tumor progression (48, 49).

The data of the current study are the first to describe the relative mRNA expression of five osteopontin splice variants in primary and metastatic melanoma tissue samples. We found that the expression levels of *OPNa, OPNb*, and *OPNc* were significantly higher in the metastatic lesions compared to the primary tumors, and *OPNc* was significantly positively correlated with increasing Breslow thickness in the primary tumors. The expression of the recently described *OPN4* and *OPN5* isoforms was shown to be downregulated in the evaluated melanoma subtypes, and *OPN4* exhibited a significant negative correlation with Breslow thickness. The relative expression of eight integrins was very low; only *ITGB3* showed detectable expression in metastatic tumors compared to the primary lesions; moreover, *ITGA2* showed significant negative correlation with the Breslow thickness of the primary tumors. Our data show that high expression of *OPNa, OPNb*, and *OPNc* is associated with poor prognosis, and *OPN4* and *ITGA2* may have an opposite role in melanoma progression. Nevertheless, further studies are needed to more specifically characterize the involvement of osteopontin splice variants in malignant melanoma progression and their interaction with integrins in cancer.

## Data Availability

The original data presented in this study are included in the article as well as in the Supplementary Material, further inquiries can be directed to the corresponding author.

## Supplementary Material

Supplementary Figure S1 Relative mRNA expression of ITGA2 gene in malignant melanoma tissue samples from tumors with different Clark stages: earlier stages (II–III. *n* = 13) and later stages (IV–V. *n* = 17).

Supplementary Figure S2 Graphs illustrating the significant positive correlations (Spearman’s rho) between expression of OPN4 and integrin genes (*ITGB3*, *ITGA5*, *ITGA9*, *ITGAV*, *ITGB1*, *ITGA3*, and *ITGA6*).

Supplementary Figure S3 Graphs illustrating the significant positive correlations (Spearman’s rho) between the expression of *OPN5* and integrin genes (*ITGA2*, *ITGAV*, *ITGB1*, *ITGA3*, and *ITGA6*).

Supplementary Figure S4 Graphs illustrating the significant negative correlations (Spearman’s rho) between the gene expression of *ITGA2* and osteopontin splice variants (*OPNa*, *OPNb*, *OPNc*).

Supplementary Figure S5 Graphs illustrating the significant negative correlation (Spearman’s rho) between the expression of ITGA6 and OPNc.
