# Supplementary material for: Gene Expression Patterns of Osteopontin Isoforms and Integrins in Malignant Melanoma
Source: Pathol Oncol Res. 2022 Aug 24;28:1610608. doi: 10.3389/pore.2022.1610608 (PMC9448871; doi:10.3389/pore.2022.1610608)
Supplement: Supplementary file 1 [file Table1.docx]

**Supplementary Table 1.** Median values of *OPN* variants mRNA expression levels (log2 transformed data) in different subtypes of malignant melanoma tissue samples

| **Median** | | | | | |
| --- | --- | --- | --- | --- | --- |
| **Melanoma subtypes** | ***OPNa*** | ***OPNb*** | ***OPNc*** | ***OPN4*** | ***OPN5*** |
| **SSM (n = 21)** | -0.350 | -1.315 | -0.410 | -1.337 | -2.578 |
| **NM (n = 10)** | 0.965 | 0.572 | -0.293 | -1.758 | -4.503 |
| **Metastasis (n = 10)** | 3.925 | 3.043 | 3.060 | 0.439 | -3.286 |
| ***p* value** | **≤0.01** | **≤0.001** | **≤0.01** | ns | ns |

SSM: superficial spreading melanoma; NM: nodular melanoma; ns: not significant
