# Supplementary figures and images for: Gene Expression Patterns of Osteopontin Isoforms and Integrins in Malignant Melanoma
Source: Pathol Oncol Res. 2022 Aug 24;28:1610608. doi: 10.3389/pore.2022.1610608 (PMC9448871; doi:10.3389/pore.2022.1610608)

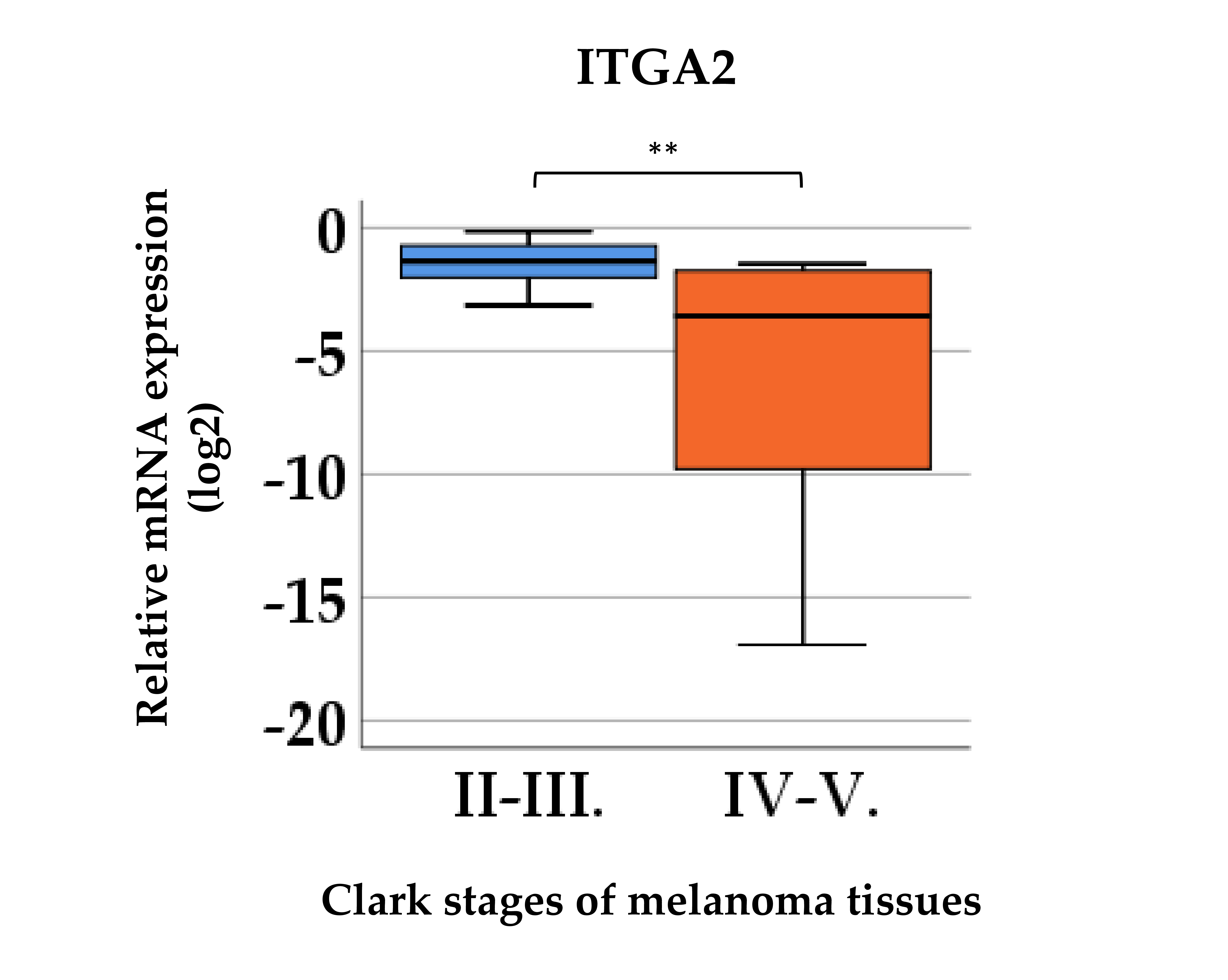

Supplement: Supplementary file 2 [file DataSheet1.zip › 1610608_Supplementary figure 1.tiff]

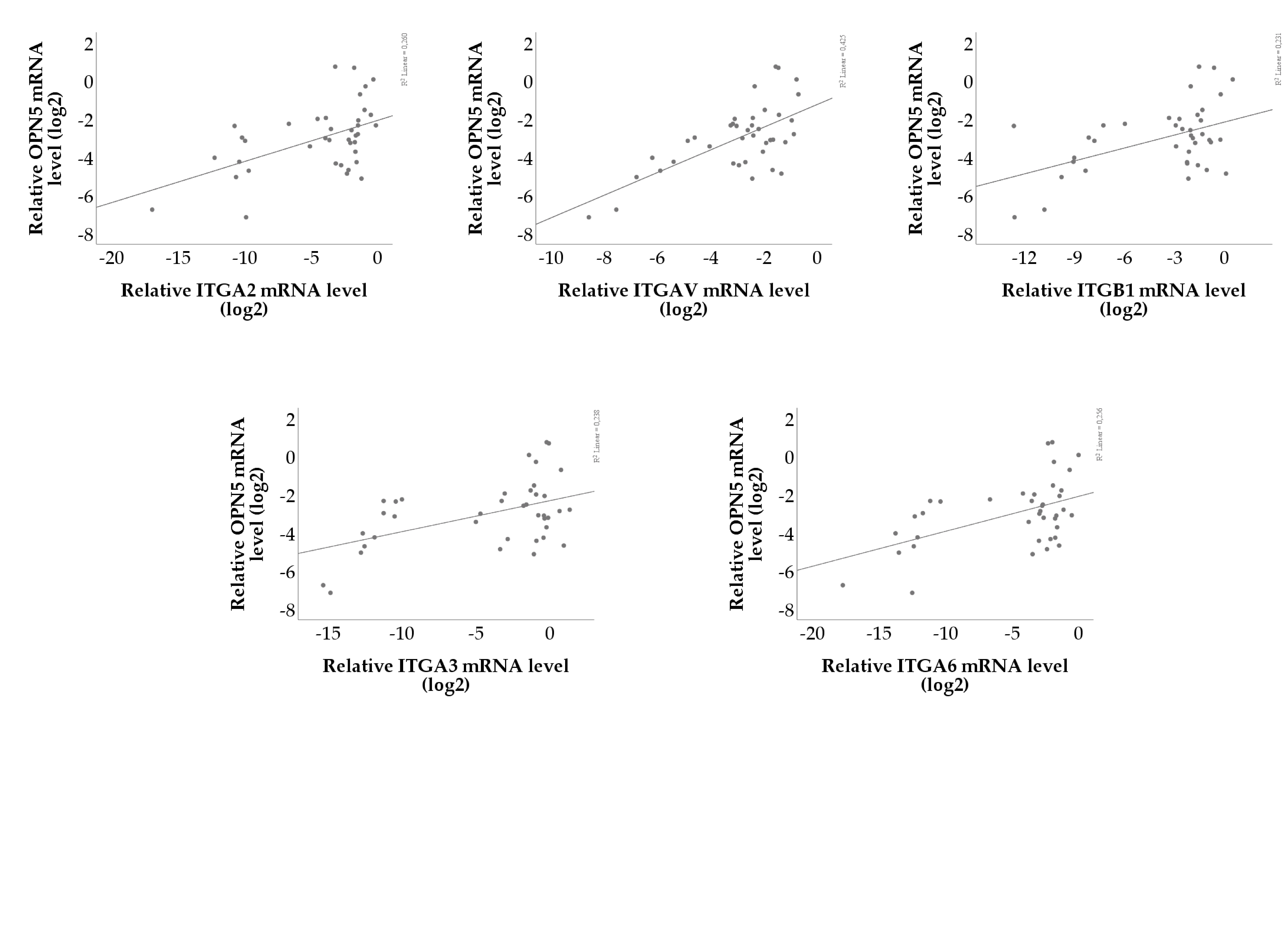

Supplement: Supplementary file 2 [file DataSheet1.zip › 1610608_Supplementary figure 3.tiff]

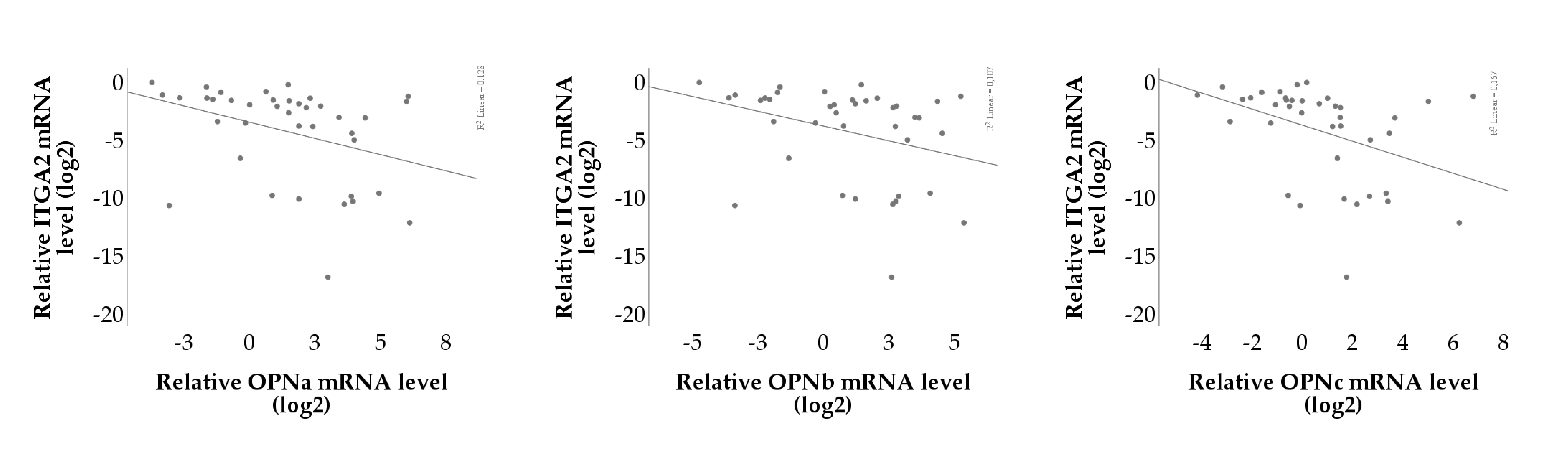

Supplement: Supplementary file 2 [file DataSheet1.zip › 1610608_Supplementary figure 4.tiff]

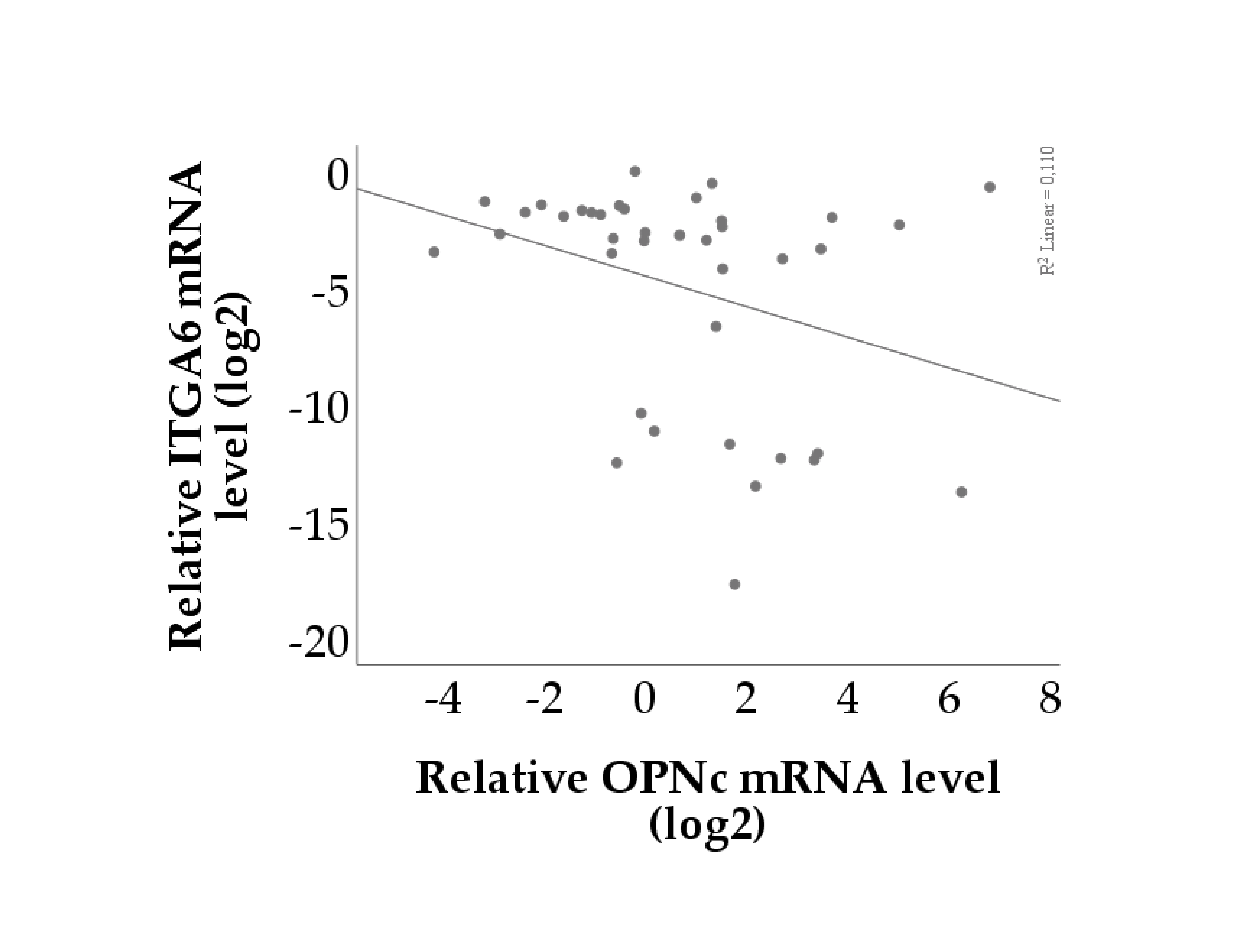

Supplement: Supplementary file 2 [file DataSheet1.zip › 1610608_Supplementary figure 5.tiff]
