# Supplementary material for: Gene Expression Patterns of Osteopontin Isoforms and Integrins in Malignant Melanoma
Source: Pathol Oncol Res. 2022 Aug 24;28:1610608. doi: 10.3389/pore.2022.1610608 (PMC9448871; doi:10.3389/pore.2022.1610608)
Supplement: Supplementary file 3 [file Table2.docx]

**Supplementary Table 2.** Median values of *OPN* variants mRNA expression levels (log2 transformed data) in malignant melanoma tissue samples with distinct Breslow thickness

| **Median** | | | | | |
| --- | --- | --- | --- | --- | --- |
| **Breslow thickness** | ***OPNa*** | ***OPNb*** | ***OPNc*** | ***OPN4*** | ***OPN5*** |
| **< 2 mm (n = 8)** | -1.24 | -1.70 | -1.24 | -1.67 | -2.05 |
| **2**–**4 mm (n = 14)** | 0.46 | 0.33 | -0.57 | -1.07 | -3.09 |
| **> 4 mm (n = 9)** | 1.50 | 0.73 | 1.40 | -2.21 | -3.71 |
| ***p* value** | ns | ns | **≤ 0.01** | ns | ns |

ns: not significant
