# Supplementary material for: Gene Expression Patterns of Osteopontin Isoforms and Integrins in Malignant Melanoma
Source: Pathol Oncol Res. 2022 Aug 24;28:1610608. doi: 10.3389/pore.2022.1610608 (PMC9448871; doi:10.3389/pore.2022.1610608)
Supplement: Supplementary file 4 [file Table5.docx]

**Supplementary Table 5.** Spearman’s rho correlation of the expression data of OPN variants and integrins. Blue color indicates the significant negative correlation, yellow indicates the significant positive correlation

| Spearman's rho | | ITGB3 | ITGA5 | ITGA2 | ITGA9 | ITGAV | ITGB1 | ITGA3 | ITGA6 |
| --- | --- | --- | --- | --- | --- | --- | --- | --- | --- |
| OPNa | Correlation Coefficient | -0.088 | -0.136 | **-0.480^**^** | -0.116 | -0.182 | -0.008 | -0.108 | -0.213 |
|  | Sig. (2-tailed) | 0.594 | 0.410 | 0.002 | 0.481 | 0.267 | 0.960 | 0.513 | 0.192 |
|  | N | 39 | 39 | 39 | 39 | 39 | 39 | 39 | 39 |
| OPNb | Correlation Coefficient | -0.052 | -0.121 | **-0.420^**^** | -0.055 | -0.127 | 0.059 | -0.084 | -0.153 |
|  | Sig. (2-tailed) | 0.754 | 0.462 | 0.008 | 0.741 | 0.440 | 0.721 | 0.610 | 0.353 |
|  | N | 39 | 39 | 39 | 39 | 39 | 39 | 39 | 39 |
| OPNc | Correlation Coefficient | -0.134 | -0.264 | -0.542^**^ | -0.272 | -0.308 | -0.201 | -0.272 | -0.398^*^ |
|  | Sig. (2-tailed) | 0.416 | 0.104 | 0.0004 | 0.094 | 0.056 | 0.221 | 0.094 | 0.012 |
|  | N | 39 | 39 | 39 | 39 | 39 | 39 | 39 | 39 |
| OPN4 | Correlation Coefficient | **0.607^**^** | **0.531^**^** | 0.283 | **0.532^**^** | **0.520^**^** | **0.591^**^** | **0.581^**^** | **0.500^**^** |
|  | Sig. (2-tailed) | 0.00004 | 0.001 | 0.080 | 0.0005 | 0.001 | 0.0001 | 0.0001 | 0.001 |
|  | N | 39 | 39 | 39 | 39 | 39 | 39 | 39 | 39 |
| OPN5 | Correlation Coefficient | 0.279 | 0.285 | **0.452^**^** | 0.248 | **0.503^**^** | **0.349^*^** | **0.365^*^** | **0.405^*^** |
|  | Sig. (2-tailed) | 0.086 | 0.079 | 0.004 | 0.129 | 0.001 | 0.030 | 0.022 | 0.010 |
|  | N | 39 | 39 | 39 | 39 | 39 | 39 | 39 | 39 |
