# Supplementary material for: Gene Expression Patterns of Osteopontin Isoforms and Integrins in Malignant Melanoma
Source: Pathol Oncol Res. 2022 Aug 24;28:1610608. doi: 10.3389/pore.2022.1610608 (PMC9448871; doi:10.3389/pore.2022.1610608)
Supplement: Supplementary file 5 [file Table3.docx]

**Supplementary Table 3.** Median values of mRNA expression levels (log2 transformed data) of eight integrins in different subtypes of malignant melanoma tissue samples. (SSM: superficial spreading melanoma; NM: nodular melanoma)

| Median | | | | | | | | |
| --- | --- | --- | --- | --- | --- | --- | --- | --- |
| Melanoma subtypes | ***ITGA2*** | ***ITGA3*** | ***ITGA5*** | ***ITGA6*** | ***ITGA9*** | ***ITGAV*** | ***ITGB1*** | ***ITGB3*** |
| SSM (n = 20) | -1.622 | -1.072 | -1.134 | -2.660 | -3.796 | -2.376 | -2.022 | 0.168 |
| NM (n = 9) | -3.630 | -2.853 | -2.383 | -2.305 | -3.257 | -3.029 | -2.218 | 1.274 |
| Metastasis (n = 10) | -4.807 | -4.843 | -1.770 | -3.539 | -4.337 | -3.570 | -2.791 | -0.863 |
| *p* value | **≤ 0.01** | ns | ns | ns | ns | ns | ns | ns |
| SSM: superficial spreading melanoma; NM: nodular melanoma, ns: not significant | | | | | | | | |
