# Supplementary material for: Gene Expression Patterns of Osteopontin Isoforms and Integrins in Malignant Melanoma
Source: Pathol Oncol Res. 2022 Aug 24;28:1610608. doi: 10.3389/pore.2022.1610608 (PMC9448871; doi:10.3389/pore.2022.1610608)
Supplement: Supplementary file 6 [file Table4.docx]

**Supplementary Table 4.** Median values of integrin mRNA expression levels (log2 transformed data) in malignant melanoma tissue samples with distinct Breslow thickness.

| Median | | | | | | | | |
| --- | --- | --- | --- | --- | --- | --- | --- | --- |
| Breslow thickness | ***ITGA2*** | ***ITGA3*** | ***ITGA5*** | ***ITGA6*** | ***ITGA9*** | ***ITGAV*** | ***ITGB1*** | ***ITGB3*** |
| < 2 mm (n = 8) | -1.11 | -1.07 | -1.54 | -1.89 | -3.70 | -2.40 | -2.02 | -1.19 |
| 2–4 mm (n = 12) | -1.89 | -0.59 | -0.99 | -1.94 | -3.30 | -1.84 | -1.67 | 2.01 |
| > 4 mm (n = 9) | -9.681 | -10.409 | -3.274 | -10.357 | -8.392 | -3.168 | -8.131 | 0.427 |
| *p* value | **≤ 0.01** | ns | ns | **≤ 0.05** | **≤ 0.01** | **≤ 0.05** | **≤ 0.05** | **≤ 0.01** |
| SSM: superficial spreading melanoma; NM: nodular melanoma; ns: not significant | | | | | | | | |
